# Multi-camera BEV video-surveillance system for efficient monitoring of social distancing

**DOI:** 10.1007/s11042-023-14416-y

**Published:** 2023-03-07

**Authors:** David Montero, Nerea Aranjuelo, Peter Leskovsky, Estíbaliz Loyo, Marcos Nieto, Naiara Aginako

**Affiliations:** 1 Computer Vision and Artificial Inteligence, University of the Basque Country, Donostia, 20018 Guipuzcoa Spain; 2 ITS and Engineering, Vicomtech, Donostia, 20009 Guipuzcoa Spain

**Keywords:** Crowd monitoring, Multi-camera tracking, 2D-3D projection, COVID-19

## Abstract

The current sanitary emergency situation caused by COVID-19 has increased the interest in controlling the flow of people in indoor infrastructures, to ensure compliance with the established security measures. Top view camera-based solutions have proven to be an effective and non-invasive approach to accomplish this task. Nevertheless, current solutions suffer from scalability problems: they cover limited range areas to avoid dealing with occlusions and only work with single camera scenarios. To overcome these problems, we present an efficient and scalable people flow monitoring system that relies on three main pillars: an optimized top view human detection neural network based on YOLO-V4, capable of working with data from cameras at different heights; a multi-camera 3D detection projection and fusion procedure, which uses the camera calibration parameters for an accurate real-world positioning; and a tracking algorithm which jointly processes the 3D detections coming from all the cameras, allowing the traceability of individuals across the entire infrastructure. The conducted experiments show that the proposed system generates robust performance indicators and that it is suitable for real-time applications to control sanitary measures in large infrastructures. Furthermore, the proposed projection approach achieves an average positioning error below 0.2 meters, with an improvement of more than 4 times compared to other methods.

## Introduction

**Fig. 1:**
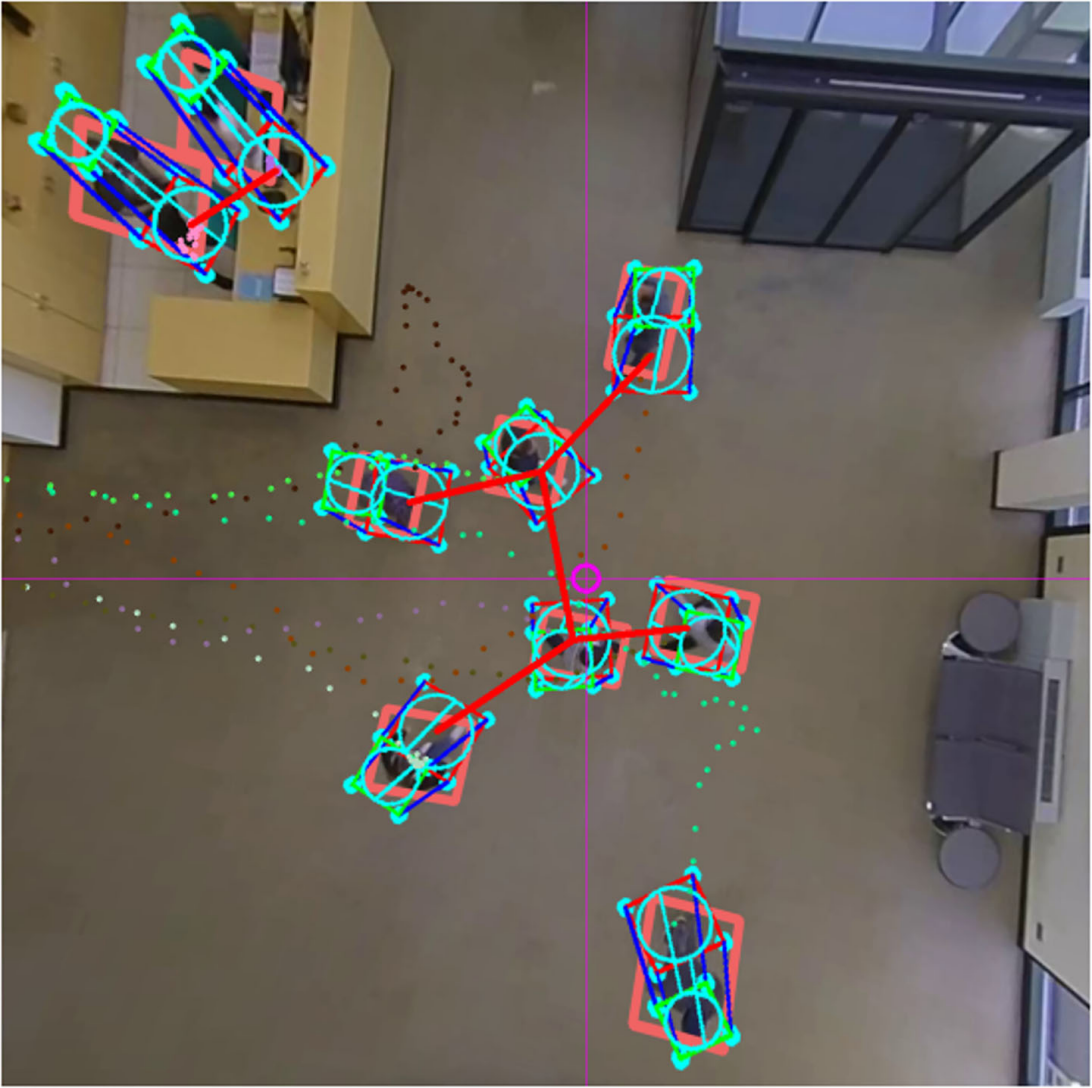
Visual example of the proposed system operation. For each 2D detection, the best fitting 3D cylinder is estimated in real-world coordinates. After a 3D tracking step, the desired metrics are computed

**Fig. 2:**
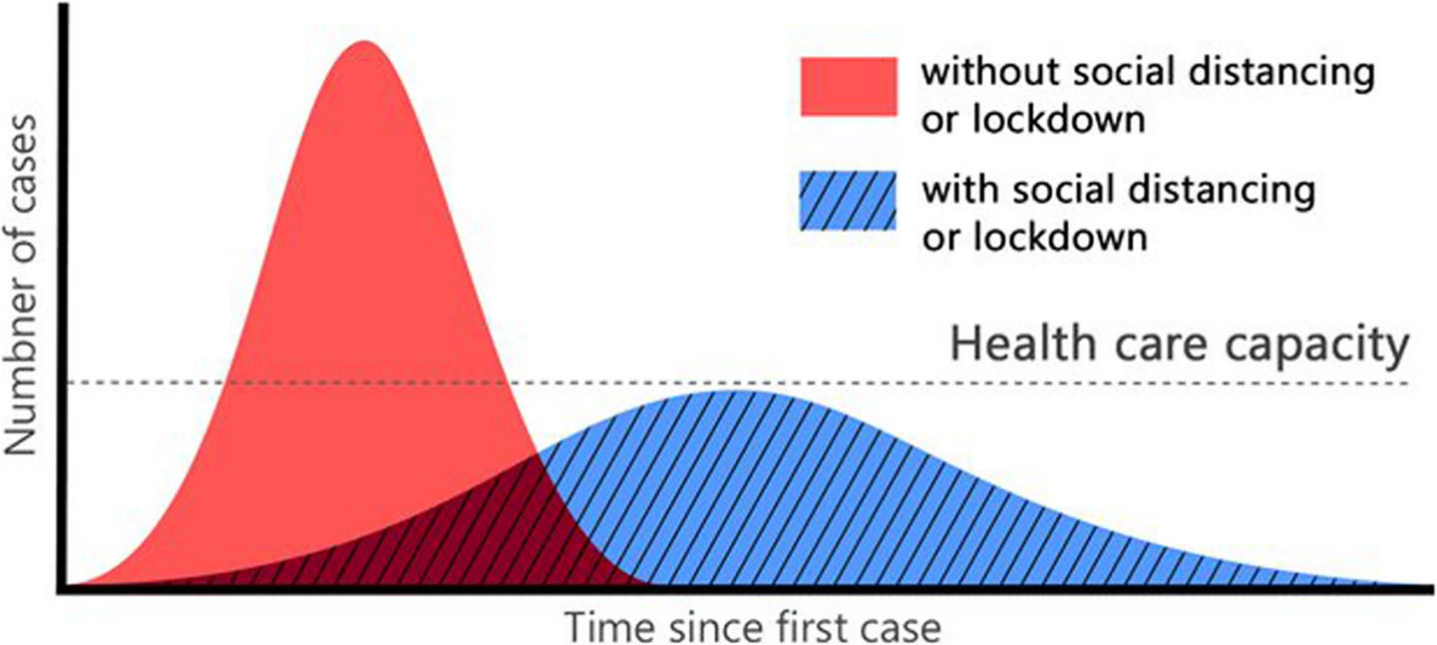
Gaussian distribution of infection transmission rate for a given population, with and without social distancing obligation. This figure was originally presented in [30]

Since the rise of COVID-19 in December 2019, numerous studies have emerged to help stop the disease spreading, tackling the problem from different perspectives [12, 27]. Most of these studies focus on preventing the spread of the virus by proposing health and social measures, and methods to ensure its compliance. Among these prevention measures, like improving the ventilation in indoor areas [1] and using medical masks [35, 36], one key measure that has been adopted by all the governments is social distancing (Fig. 1). Recent research has confirmed the evidence that maintaining a social distance of 1.6 to 2 meters highly reduces the disease spreading [38, 39], as shown in Fig. 2.

Thus, an important need has arisen to create applications that are capable of monitoring people in indoor and outdoor infrastructures to guarantee a safe interpersonal distance, respect the indoor capacity limitation, know the most crowded time intervals or track subjects who violate the established measures.

A full variety of solutions have been proposed, addressing the problem from different perspectives, such as using wi-fi signals [40], wearable devices [10], drones [33], mobile robots [31], etc.

Among all these methodologies, camera-based solutions have proven to be an effective, non-invasive and affordable alternative to accomplish this task. Furthermore, these methods can take advantage of the camera infrastructure already available in smart cities, facilitating their scalability and sustainability. Within the camera-based solutions, we find different approaches, such as face recognition [7, 14, 22] or crowd density estimation [13, 41, 46]. However, for the considered use case, the approach that works best is the one that combines human detection and tracking [25, 26, 30, 34, 37, 43], since it allows covering wider areas than with face recognition, and makes it possible to collect spatio-temporal information about the individuals who are violating the measures (unlike solutions based on density estimation). Nevertheless, most of the systems based on this methodology only work with side or frontal view perspectives. In this setup, cameras still produce an important amount of occlusions, especially when dealing with very large and crowded surfaces (e.g. airports or shopping centers). These occlusions reduce the accuracy of the tracking algorithms and of the 3D detection projection, which are crucial for computing COVID-19-related performance indicators (PIs), such as the interpersonal distance or the indoor capacity limitation.

These problems can be mitigated by using algorithms that detect people on the Bird’s-Eye-View (BEV) domain and omnidirectional cameras. Thus, the number of occlusions are minimized and the covered area is maximized, as the cameras are placed in the ceiling. Therefore, the top-view perspective makes this approach suitable for applications related with the compliment of the COVID-19 sanitary measures. Although there is some recent research [4, 6], this topic still remains underexplored, as the proposed solutions only work with single-camera scenarios and for very limited monitoring areas.

For this reason, we present a multi-camera BEV people flow monitoring system, capable of extracting reliable real-time PIs in extremely large infrastructures, such as airports or shopping centers. The proposed system relies on three main pillars: an optimized top view human detection neural network based on YOLO-V4, capable of working with data from cameras at different heights; a multi-camera 3D detection projection and fusion procedure, which uses the camera calibration parameters for an accurate real-world positioning; and a tracking algorithm which jointly processes the 3D detections coming from all the cameras.

We highlight two novel contributions in this work:

- A modification in the traditional pipeline that allows our system to operate efficiently in multi-camera environments. Unlike the rest of the proposed methods, we move the projection step to real world coordinates just after the detection step for each camera (instead of applying it after the tracking step). Then, thanks to an initial multi-camera calibration procedure, we are able to track the subjects uninterruptedly all over the monitored area using just a single tracker instance for processing all the detections. Furthermore, this approach allows using cameras installed at different heights, since it does not take into account the detection bounding box for the multi-camera fusion but the real position in meters.
- A 3D projection and multi-camera fusion procedure. Using the intrinsic and extrinsic parameters of the involved cameras, it estimates the best fitting 3D cylinder for each detected bounding box and fuses the cylinders of the overlapping regions of the camera views that belong to the same person. This corrects possible occlusion problems and allows us to expand the useful range of the cameras.

We conduct different experiments to demonstrate that the proposed system generates robust PIs and that it is suitable for real-time applications to control sanitary measures, such as guaranteeing a safe interpersonal distance, respecting the indoor capacity limitations, identifying the most crowded time intervals or tracking subjects who violate the established measures. Furthermore, the proposed projection approach achieves an average positioning error below 0.2 meters, with an improvement of more than 4 times compared to other methods. An example of the proposed system operation is shown in Fig. 1.

The rest of the paper is organized as follows. First, we present a review of the related work in Section 2. Section 3 describes the proposed method. In Section 4 we provide experimental results. A discussion about the method and the results is presented in Section 5 Finally, conclusions are given in Section 6.

## Related work

### Social distance monitoring

In recent months, several methods aiming to monitor compliance with sanitary measures have been proposed, specially for social distance monitoring [23, 24], addressing the problem from different perspectives. For example, in [31] the authors developed a mobile robot for social distance monitoring in crowded scenarios. It was equipped with an RGB-D camera and a 2D lidar to make collision-free navigation in mass gatherings. They used YOLO-V3 Deep Neural Network (DNN) [29] along with Deep SORT algorithm [42] for detection and tracking of individuals, respectively. However, the limited field of view of the robot and the cost of acquiring and maintaining several robots make this solution unsuitable for large infrastructures. In [33], the authors use a drone to deploy a social distance monitoring system. The drone detect human heads in realtime and then calculate the social distancing between pedestrians on UAV images using a DNN that follows the PeleeNet as backbone and further incorporates the multi-scale features and spatial attention to enhance the features of small objects. In [28], another drone-based method for social distance monitoring was proposed. Relying on the drone’s camera and YOLO-V3 algorithm, the system was able to detect people from side or frontal-view images and to monitor if the social distance was respected and if subjects were wearing masks. Nevertheless, these drone-based solutions are only valid for outdoor environments and they have a high associated cost due to the drone acquisition and maintenance. On the other hand, in [10] the authors used a wearable, oscillating magnetic field-based proximity device for social distance monitoring. Despite this solution achieves excellent results in indoor and outdoor environments, it is unfeasible as it requires all subjects to wear the device.

Among all these innovative solutions, systems using vision-based human detection have proven to be the best value for money, as they only need a monocular camera and a GPU-enabled server for real-time people monitoring and they can cover wide areas. Furthermore, they are also less intrusive than other methods mentioned before. Rezaei and Azarmi [30] proposed a pedestrian-detection-based social distance monitoring system. Using the YOLO-V4 [11] model pretrained with COCO dataset and SORT tracking algorithm [9] they were able to operate accurately in real-time. In [26] and [43], the authors also proposed a social distance monitoring system based on YOLO-V3 DNN with Deep SORT tracking algorithm and YOLO-V4 respectively. Su et al. [37] follow the same pipeline and combine the euclidean distance with spatio-temporal information about the trajectory of the pedestrians to better understand the scene. Shorfuzzaman et al. [34] propose to add a perspective transformation to bird-eye-view to determine the ROI in which social distancing will be monitored, but they do not add a tracking step. However, all these systems suffer from the same problem. Pedestrian occlusions are very common when dealing with large or crowded scenarios and using frontal or side-view images.

This problem can be mitigated by using a BEV perspective with omnidirectional cameras. Thus, the occlusions are minimized in the central area of the camera and the covered area is maximized. This approach was adopted in [6] and [4]. Nevertheless, their proposed systems works only with the central area of a single camera, where the occlusions need not to be considered. Therefore, for covering wide areas, an important number of cameras would be needed. This increases the hardware requirements and the cost of the system, and makes it unsuitable for large infrastructures. Furthermore, the question of joining tracks across camera views would have to be addressed too.

To overcome these problems, our proposed multi-camera BEV people flow monitoring system uses a multi-camera detection fusion procedure. The 2D detections received from the detection DNN are projected to real-world coordinates and the best-fitting 3D cylinder is estimated for every given detection. Then, the detections of the overlapped cameras are fused, correcting possible occlusion problems and allowing us to expand the useful range of the cameras. Finally, the 3D trajectories are effectively computed by our online 3D version of the tracking algorithm proposed in [21]. This way, we only need to use a single tracker for all the cameras to track the subjects over the entire monitored area, no matter in which camera’s view is detected.

### Overhead human detection-based tracking

In recent years, several tracking algorithms have been proposed to deal with overhead people detections. Ahmed and Adnan [3], proposed rHOG, an overhead tracking algorithm which uses the variable size bounding boxes with different orientations, with respect to the radial distance of the center of the image. In [5], the authors proposed a people tracking algorithm for industrial environments that works with motion blobs gathered by an overhead camera. This algorithm, based on rHOG, uses the history of already imaged population with the anticipated blob position of the person observed. Other works base their algorithms on Kalman [2] or particle filters [16].

Although these algorithms work well with a single camera, they are not suitable for multi-camera scenarios, as they are not able to merge data coming from multiple overlapped cameras. We could add a fusion stage after the tracking process, but that would require having multiple tracking instances calibrated for each camera, which is inefficient and complicates the system installation process.

For this reason, we propose to alter the order of the stages: first, to project all the detections in the real world and combine the ones in the overlapping areas and, then, to apply a single 3D tracking process. Thus, we adapt the 3D offline algorithm presented in [21] for the considered use case, resulting in an online version optimized to work with human detections described in Section 3.7.

## Proposed method

### Problem definition

We consider the problem of efficient monitoring of a set of established security measures in large infrastructures using non-invasive technology. More specifically, we aim to create a video-surveillance system capable of monitoring compliance with social distance and capacity limitation measures, as well as tracking the offenders or the possibly infected subjects. Therefore, the system must be capable of merging the information coming from multiple cameras to track subjects all over the monitored region. We use overlapped camera views to track people across views.

**Fig. 3:**
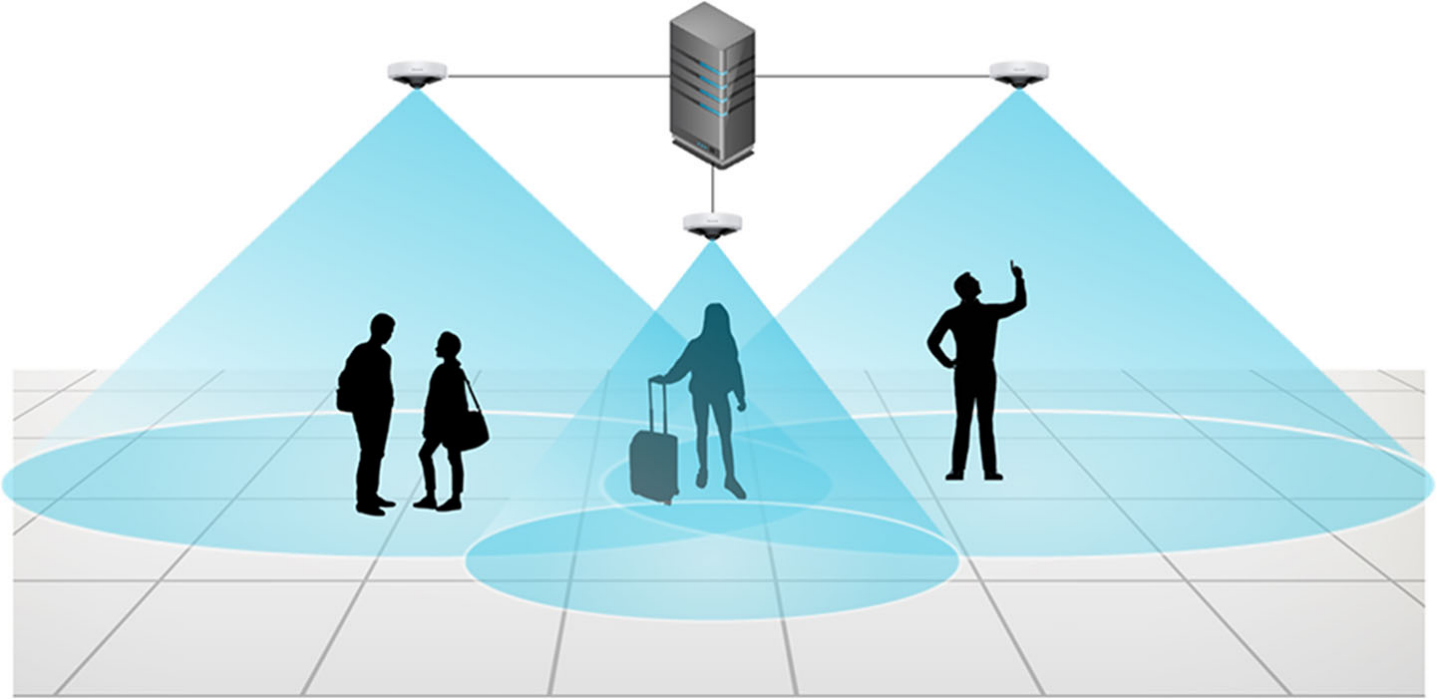
Illustration of the considered scenario. Multiple omnidirectional cameras with a small overlap cover the monitored area. The cameras are connected to a central GPU-enable server

For this task, the system will extract the necessary information from a set of cameras placed on the ceiling of the monitored infrastructure. The number of cameras depends on the area to be covered and on the height of the ceiling. The set up needs to guarantee that all the areas of interest are visible by the cameras with a minimum resolution (limited by the capabilities of the human detection network). The omnidirectional cameras with fish-eye lenses have a wide field of view, which makes them appropriate for monitoring large areas with a minimum number of sensors. Intrinsic and extrinsic parameters of all the cameras need to be available for an accurate distance measuring. An image illustrating the considered use case is presented in Fig. 3. The system must be able to report reliable and real-time information about the state of measures compliance using minimum processing requirements, preferably a single-GPU server.

### System overview

**Fig. 4:**
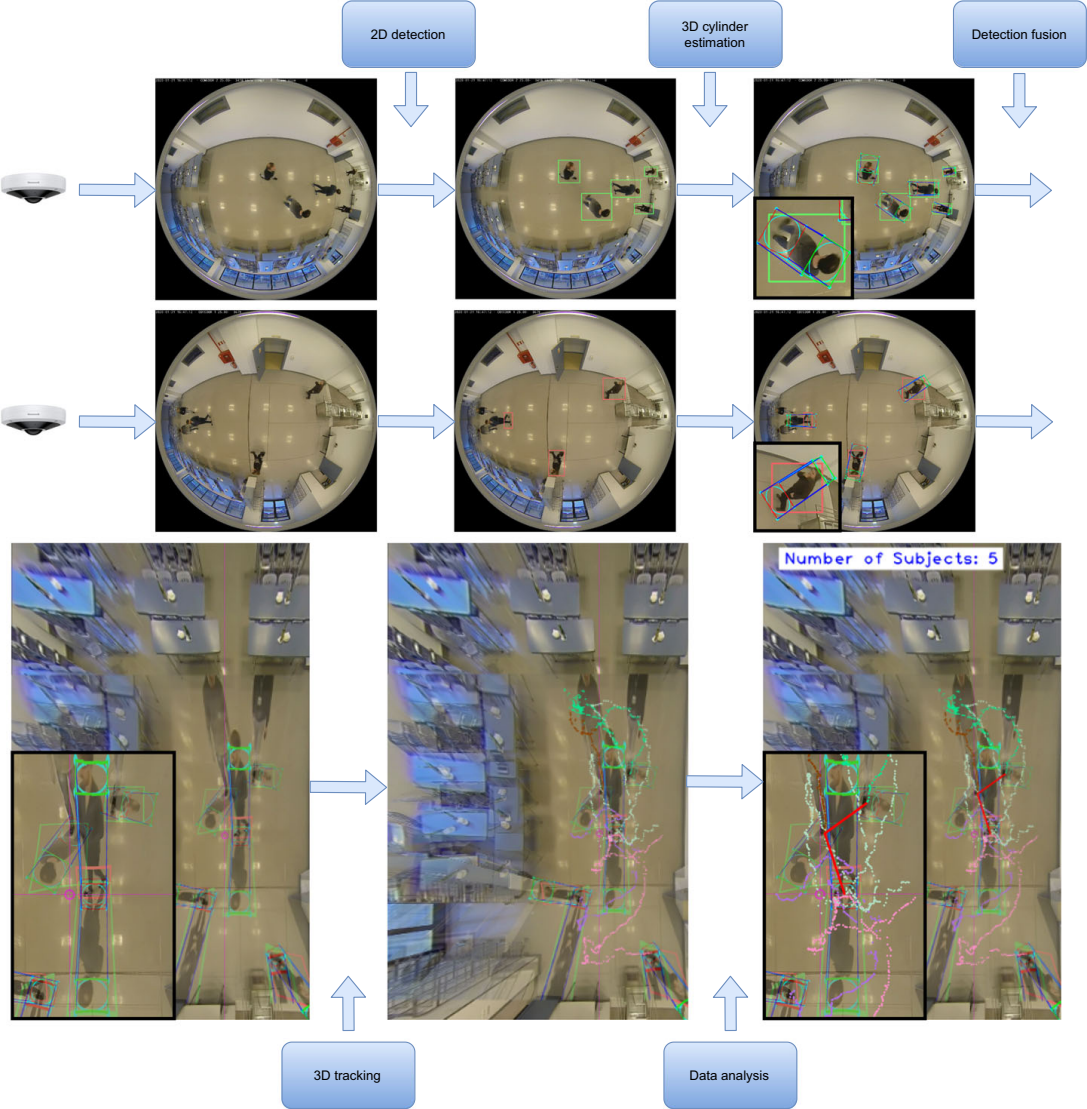
Overview graphical diagram of the proposed workflow. Note that the rectified images (in the lower row) are generated only for visualization purposes and are not necessary for fusing the detections, tracking or data analysis

**Fig. 5:**
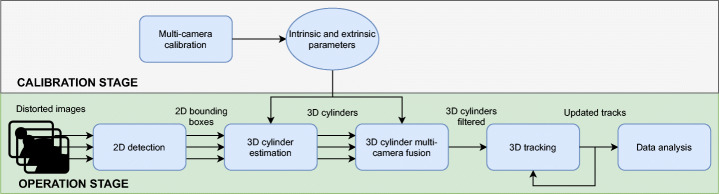
Overview flowchart of the proposed workflow. Note that until the cylinders fusion step, the detections of each camera are processed separately

An overview graphical diagram of the proposed workflow is shown in Fig. 4 and a flowchart in Fig. 5.

The system requires an initial camera calibration process (Section 3.3). First, the images are grabbed from each configured camera. Then, they are preprocessed in parallel using CUDA library and fed into the pruned version of YOLO-V4 detector, implemented in TensorRT framework (Section 3.4). Therefore, from the original (distorted) images, a set of detections is obtained for each camera *c* at each frame *t*, $\mathcal {D}_{c}=\{D_{c,t,i}\}$. Each detection is modeled as a 4-point rectangle in image coordinates *D* = (*x*_0_,*y*_0_,*x*_1_,*y*_1_,*x*_2_,*y*_2_,*x*_3_,*y*_3_).

As discussed in Section 2.2, we alter the typical order of the tracking and projection stages for two reasons: it is easier to estimate the trajectory using world coordinates than image coordinates from an omnidirectional camera (where the bounding box varies rapidly); and we only need a single tracker instance to process all detections in world coordinates. Thus, from detections $\mathcal {D}_{c}$ a fitting process yields the desired 3D cylinder shapes $\mathcal {C}_{c}=\{C_{c,t,i}\}$, where each cylinder is encoded as *C* = (*X*,*Y*,*H*,*r*), where (*X*,*Y* ) is the center point in the XY plane and (*H*,*r*) represent its height and radius, respectively. Fitting process is explained in Section 3.5. Then, a fusion mechanism determines which cylinders correspond to the same object for cameras with overlapped fields of view. Let’s denote fused cylinders as *C*, where we have removed the c sub-index, as now all cylinders are expressed in global 3D coordinates and not related anymore to any specific camera. Fusion procedure is presented in Section 3.6. Next, the tracking stage takes these cylinders, applying a constant-velocity predicting model, plus managing appearance and disappearance of objects, miss-detections, etc. The tracking mechanism is explained in Section 3.7. Note that tracks are expressed as follows: $\mathcal {T}=\{T_{k}\}$, where $T=(X, Y, H, r, \dot {X}, \dot {Y}, \ddot {X}, \ddot {Y})$ to account for the derivative dimensions for prediction phase. Finally, the output time-consistent tracks are analysed to extract the necessary information for the monitoring of the established security measures (Section 3.8).

### System calibration

When the system is set up, and prior to the first operation, a calibration step is needed in order to compute the intrinsic and extrinsic parameters. The estimated camera parameters are used for mapping the image coordinates to the 3D world coordinates and for locating each camera with respect to the others. Consequently, we are able not only to get the real position of each person detected in the scene but also to detect when they move from one camera to another and to merge the detections from overlapped cameras.

The well-known fish-eye camera model is used in this project, where the projection process is governed by a fish-eye distortion vector **k** = (*k*_1_,*k*_2_,*k*_3_,*k*_4_) and a linear projection matrix *K* which holds the focal length and principal point parameters. As a result of the calibration, for each camera, the intrinsic (*K*, and **k**) and extrinsic (rotation matrix *R* and translation **t**) are obtained. The pose of each camera is expressed with respect a common 3D point used as world reference.

Any 3D point in the world **X** = (*X*,*Y*,*Z*,1)^⊤^ expressed in homogeneous coordinates can then be projected into any of the images. First by representing the point with respect to the camera coordinate system, **X**_*c*_ = *P*_*c*_**X**, where *P*_*c*_ = (*R*|**t**;**0**|1) is the 4 × 4 corresponding pose matrix. The fish-eye distortion model is then applied on the 3D rays joining **X**_*c*_ and the camera optical center, by defining *a* = *X*_*c*_/*Z*_*c*_ and *b* = *Y*_*c*_/*Z*_*c*_, and *r*^2^ = *a*^2^ + *b*^2^. The longitude angle *𝜃* = *arctan*(*r*). The distortion vector **k** is then applied to obtain the angle of incidence *𝜃*_*d*_ = *𝜃*(1 + *k*_1_*𝜃*^2^ + *k*_3_*𝜃*^4^ + *k*_3_*𝜃*^6^ + *k*_4_*𝜃*^8^). The point in the normalized domain is then obtained as $(x^{\prime }, y^{\prime }) = (a\theta _{d}/r, b\theta _{d}/r)$, and its projection into the image domain as $(u, v, w) = K(x^{\prime }, y^{\prime }, 1)^{\top }$ (pixel values are obtained as *x* = *u*/*w* and *y* = *v*/*w*).

The calibration can be used as well to re-project any point in the image plane (x, y) into a 3D ray starting from the optical center of the camera, applying $\mathbf {r}=(u^{\prime }, v^{\prime }, w^{\prime })=K^{-1}(x, y, 1)^{\top }$ and normalizing so ||*r*|| = 1. Then, if a 3D world plane is selected (e.g. *Z* = 0), the intersection of the 3D ray with the plane determines a 3D point in the world. This is useful to re-project 2D image points of objects in the ground plane to obtain their position in the XY plane in world coordinates (note this assumption holds true only if the 2D image point correspond to an object or part of object which is touching or at the ground level).

Retrieving the 3D ray **r** from pixels (x, y) implies inverting the fish-eye distortion vector, which can be accomplished using iterative minimization processes (we are using OpenCV’s implementation). In addition, to speed up the re-projection process, it is recommended to create remap functions by pre-computing the mapping relation between points in the images and the 3D space, giving as a result the ability to create rectified and Bird’s-eye View (BEV) of multiple cameras in a single step.

### People detection using overhead cameras

Similar to [8], we train YOLO-V4 object detection Convolutional Neural Network (CNN) to detect people directly in overhead images from fish-eye cameras. We use this single-stage detector because it provides a good balance between accuracy and inference time. In a multi-camera system it is important to guarantee a fast inference for a real-time analysis. Compared to previous versions, YOLO-V4 includes detections at three scales, which improves the small object detection accuracy.

Our aim is to design a system capable of working with a camera installed 3 to 10 meters high, so that it is suitable for different large space scenarios. Consequently, our detector should work on this height range. Even if the YOLO-V4 model provides detections at different scales, the objects’ scale varies considerably for such a big range. In order to ensure the robustness of the model no matter the height of the camera, we add an image scaling step previous to the detection, which resizes the image to guarantee that the people size in the center of the image is stable no matter the installation height (approximately 20 × 20 pixels). In addition, we train two models, one targeted for the lowest heights (3-6 m) and another for the highest installations (6-10 m).

As there is no public dataset with top-view fish-eye images of large spaces focused on human detection and multi-camera systems, we use several recordings to build our training dataset. We set up two omnidirectional cameras installed at 3.3 meters and another camera at 8 meters. We capture 10,000 images for the lower height range and 10,000 images for the upper one. In addition, to augment both ranges’ data variety we add 5,600 synthetic images from the Advanced Synthetic Dataset presented in [8] to each of the datasets. We manually annotate the captured data. As shown in [45], rotation and histogram equalization are some of the most efficient image augmentations for training accurate object detection CNNs. Consequently, we apply rotations, flipping and histogram equalization augmentations (CLAHE) to our images. We randomly combine these augmentations and generate 4 new samples for each image. The images are resized to 512 × 512 for the models training.

We train both models on a NVIDIA Tesla P100 using Darknet framework [11]. We initialize the models with pre-trained weights on the MS COCO dataset [19] and train them for 40,000 iterations with a learning rate of 0,001 and a weight decay of 0,0005. We use the stochastic gradient descent optimizer with a batch size of 64 images.

To further increase the performance of the model we apply two optimization processes. First, we apply the weight pruning procedure described in [44]. It is an iterative process with three stages in each iteration:

- Network training penalizing the scaling weights of the batch normalization layers in the cost function.
- Network pruning percentage of convolutional filters corresponding to the lowest batch normalization scaling weights.
- Pruned network fine-tuning without penalization.

This procedure is repeated until the desired balance between precision and speed is reached. In our case, we pruned each network 3 times eliminating 50%, 50% and 70% of the remaining filters, keeping at least 10% of filters in each layer.

Finally, the pruned models are ported to TensorRT framework to apply hardware-level optimizations. With these optimizations we are able to reduce the inference time almost a 90% for each model, from 22 fps to 110 fps.

### 3D cylinder estimation

The next stage consists of transferring detections $\mathcal {D}_{c}$ from the 2D domain of all camera images to the 3D real world domain using the intrinsic and extrinsic camera parameters. More specifically, for each 2D detection *D*_*c*,*t*,*i*_ we estimate the 3D cylinder *C*_*c*,*t*,*i*_ whose projection on the image best fits the original bounding box. For this task, we adopted a Greedy Algorithm approach [20].

**Fig. 6:**
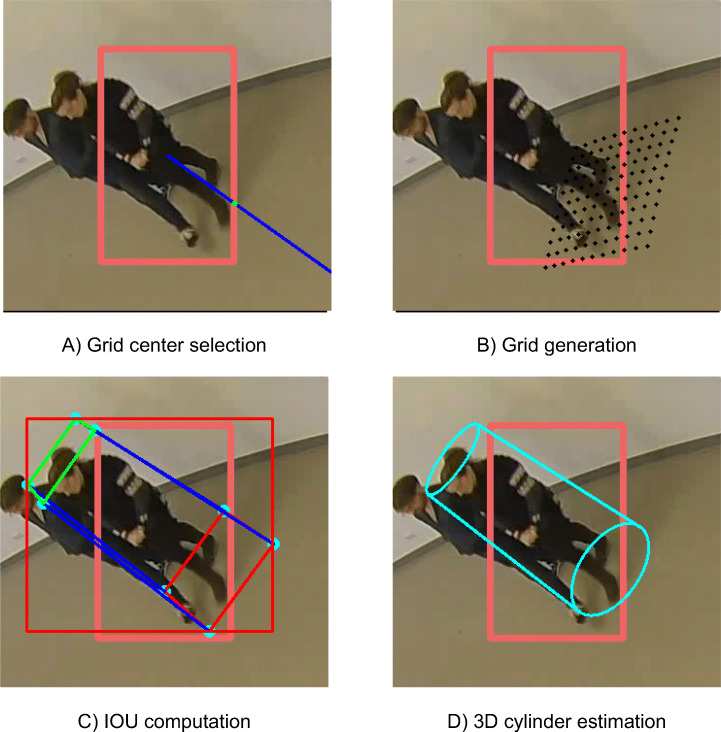
Different steps for the 3D cylinder estimation. The initial red bounding box corresponds to the 2D detection and the green point in the first image corresponds to the selected grid center

For each detection *D*_*c*,*t*,*i*_, a regular grid of 3D points $\mathcal {G}=\{G_{x,y}\}$ corresponding to possible cylinder center-points at the XY plane is generated, *G*_*x*,*y*_ = (*x*,*y*,0). The grid center is the 3D re-projection of the point of the bounding box closest to the center of the image. We choose this point because, for cameras with fish-eye lenses, the furthest point from the camera of a vertical object (i.e. the feet position of a person) corresponds to the object point closest to the center of the generated image (see Fig. 6-A). The grid is defined with two parameters: the maximum distance to the center and a distance step between points. We estimate that the maximum distance to the center of the grid cannot be greater than 0.5 meters and that a distance of 0.1 meters between points is sufficient to cover the space accurately (see Fig. 6-B).

Once $\mathcal {G}$ is defined, a set of cylinders {*C*_*k*_} is generated using each grid point as the center of the cylinder at the XY plane. For each grid point, several cylinders are created, varying their diameter (min 0.5, max 1.0, step 0.15 m), and height (min 1.5, max 1.9, step 0.2 m), creating a regular sampling of the space of the cylinder *C*_*c*,*t*,*i*_. The selected values have been chosen as a trade-off between the sampling density and the feet positioning error. In addition, the cylinder model is refined to better represent human shapes by reducing the upper circle radius by a constant factor (we have used 0.6 in our experiments) with respect to the lower circle. As a consequence, the cylinder becomes a truncated cone or frustrum. Since the factor is constant, it is not included into the state vector representations.

The fitting stage consists of a maximum likelihood estimator (MLE) process. A cost function is created to measure the likelihood of a given cylinder *C*_*j*_ = (*X*,*Y*,*H*,*r*) to fit into detection *D*_*c*,*t*,*i*_. The ideal cost function would be to project the cylinder outline points into the image and compute an IoU (Intersection over Union) value. For the sake of computational efficiency, the cylinder is simplified to its outer 3D cuboid, which is defined as 8 points *C*_*j*,*k*_, *k* = 1..8, that can be projected into image points as *c*_*j*,*k*_ = *P*_*c*_*C*_*j*,*k*_ using homogeneous coordinates (see Fig. 6-C).

Using this cost function, the MLE estimator is obtained as the weighted sum of the grid cylinders:

1 $$ C^{*}_{c,t,i}=\frac{1}{N}\sum\limits_{j=1}^{N} IoU(b\{c_{j,k}\}, D_{c,t,i}) C_{j} $$

where N is the total number of cylinders in the grid, spanning the three considered parameters (center, diameter and height), *b* is the bounding rectangle for the projected 2D points of the cuboid, and *IoU* is the Intersection Over Union function, obtaining the MLE estimator (see Fig. 6-D).

**Fig. 7:**
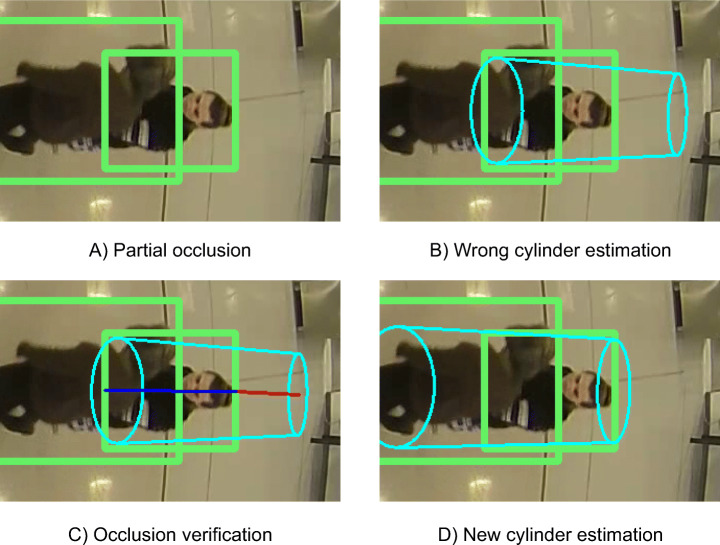
Cylinder correction from a partially-occluded detection. Note that in Figure C the distance between the points of the bounding box being furthest and closest to the image center is depicted in blue and the salient part of the cylinder is represented by the red line. The ratio between these two distances relates to the occlusion level of the detection

Although the number of occlusions is greatly reduced in BEV images from fisheye cameras, partial occlusions of the lower half of the body may appear (see Fig. 7-A). When this type of occlusions occur, the estimated cylinder $C^{*}_{c,t,i}$ is wrong and its upper part protrudes noticeably from the bounding box by the part furthest from the center of the image (see Fig. 7-B). This protrusion is measurable and thus an occlusion can then be detected if the salient part exceeds a certain threshold. We define the threshold value as the 10*%* of the distance between the points of the bounding box furthest and closest to the center of the image, which is inversely proportional to the occlusion level. Therefore, assuming that the upper point of the head, corresponding to the point of the bounding box furthest from the center of the image, is not occluded, we rebuild the cylinder from the upper center using an average human height value of 1.7 meters (see Fig. 7-D). Although the real height is likely to differ from this average height, the error produced by the occlusion is substantially reduced.

As a consequence, we can then assume that the lower center point of the person corresponds to the lower center of the estimated cylinder. In Section 4.2, we compare the proposed projection method with other approaches, such as the one used in the related works [4, 6, 26, 30, 43], consisting of projecting the center of the detection bounding box. The results show that the proposed method achieves more accurate estimations and, consequently, better results in the evaluation.

### Multi-camera detection fusion

**Fig. 8:**
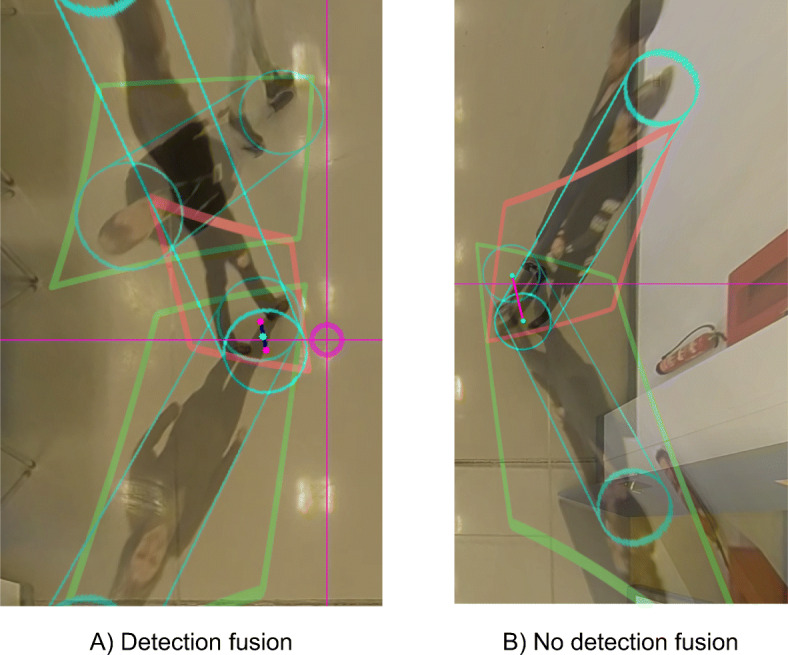
Detection fusion procedure examples. In the left image, the positions of a subject captured by two overlapped cameras are fused, as the distance between the feet points is less than the defined threshold. In the right image, the cylinders belong to different subjects occluding each other to their opposite camera. In this case, the detections are not merged, as their distance is greater than the threshold

Once all detections *D*_*c*,*t*,*i*_ have been mapped to 3D world coordinates as cylinders *C*_*c*,*t*,*i*_, we search for duplicate detections in the overlap areas between two or more cameras and fuse them. The procedure consists of selecting detections that fall inside the overlapping region, and comparing them with the detections of the other camera. Two detections from different cameras are then merged if the 3D distance in the XY plane between their center points is below a certain threshold (see Fig. 8). The threshold is defined taking into account the accuracy of the calibration parameters and the average positioning error of the detections. For our experiments we selected a threshold of 0.45 meters.

From this step onwards, cylinders are no longer attached to any particular camera, and thus treated as 3D objects *C*_*t*,*i*_ in the world coordinate system.

### 3D people tracking

The proposed 3D tracking algorithm is based on a data-association multi-object tracking approach [21]. The original algorithm was created to track cuboids belonging to different types of objects (vehicles and pedestrians). It is composed of two components: an online tracker and an offline post-process to smooth the trajectories and the shape of the cuboids. We modify the original algorithm taking into account the following three requirements: we only want to estimate the trajectory of people; the shape of the person is not important, it only matters that the position of their feet is precise; and the algorithm must be online. According to the latest requirement, we remove the offline cuboid smoothing component.

The remaining online tracker component consists of three stages: prediction, association and estimation. In the prediction stage, the value of the variables of each track *T*_*k*_ is updated based on its history using a constant-acceleration model. Such model would be useful for vehicles or even for people in scenarios where the trajectories are more steady (such as parking lots or roads). However, in scenarios such as shopping centers or airports, trajectories are more chaotic and the acceleration in one time step can vary enormously. Therefore, we decided to adopt a constant-velocity model.

During the next stage, an association matrix is created with the association likelihood between predicated tracks $\{T^{-}_{k}\}$ and detections {*C*_*t*,*i*_}. The likelihood function compares the cylinders the same way as described for the multi-camera detection fusion approach (based on the distance between the feet points of the cylinders). Finally, the estimation stage updates the state of each track fusing the positioning information of the prediction and the associated cylinder following the procedure described in the original work. In order to avoid generating erroneous tracks due to false positives in the detection stage, we do not consider a track as active until it accumulates 3 or more associated detections. In the same way, to avoid removing active tracks due to false negatives in the detection stage, we keep a track as active until it has no associated detections for 5 consecutive frames.

### Data analytics

The tracks generated in the previous stage contain all the necessary information for monitoring the compliance with the main sanitary measures against COVID-19. Moreover, this information can also be used to carry out other types of tasks, such as crowd behavior understanding [17], monitoring the entry and exit of zones, the size of the waiting queues, register the most visited stands etc. In this work we focus on three tasks:

- Social distance monitoring: for this task, the Euclidean distance between the current position of each tracked subject and that of the rest is calculated and compared with the limit established by the authorities.
- Indoor capacity limitation: the number of active tracks is checked in every time step to ensure the capacity limits are not exceeded.
- Tracking of individuals who violate the sanitary measures.

**Fig. 9:**
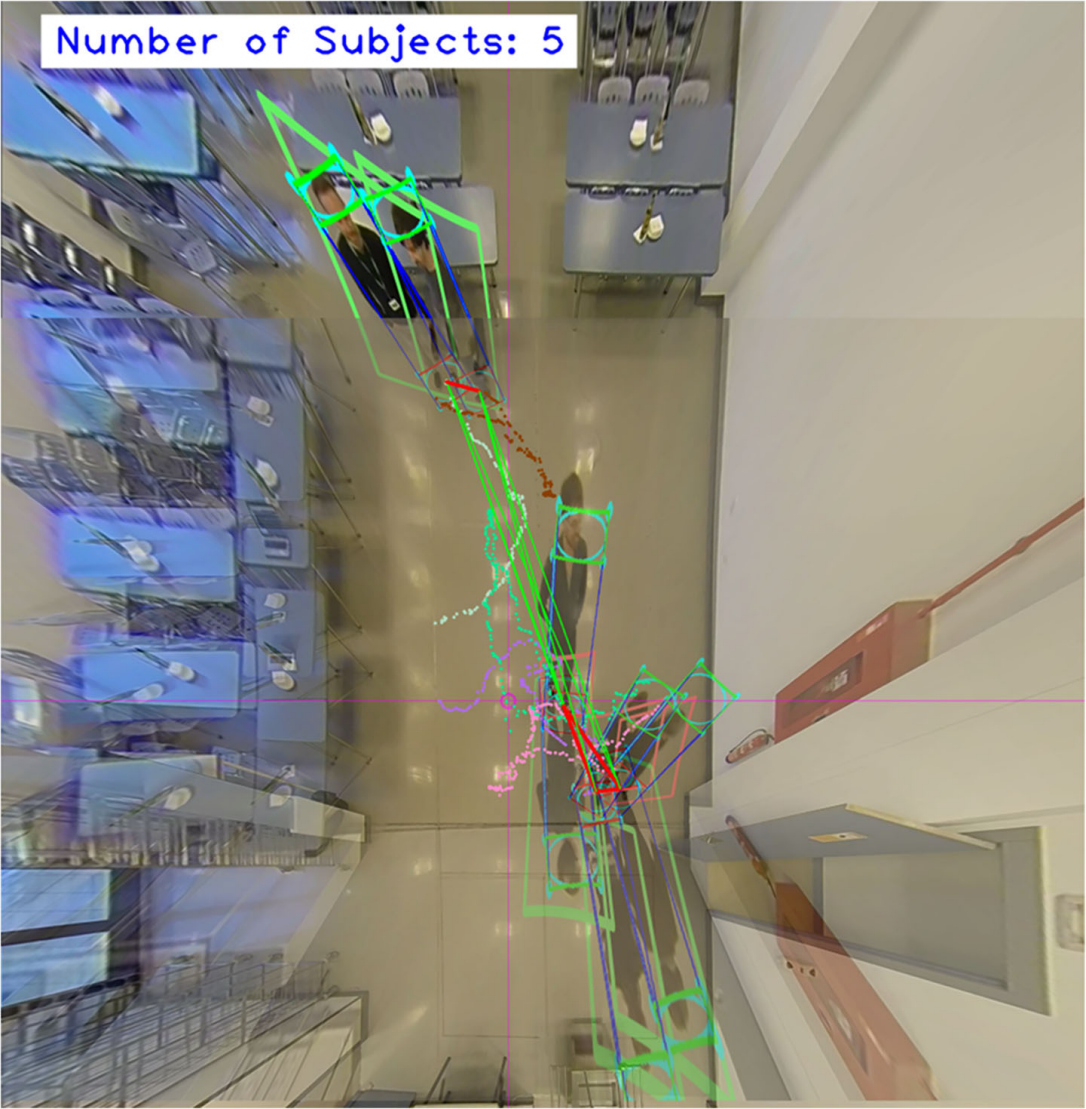
Example of the results of the data analytics procedure. The interpersonal distance is measured for every pair of tracks. The red lines means that the defined safe distance is being violated. The colored dot lines represents the trajectory of every track

An example of the results of the data analytics is presented in Fig. 9.

## Experiments

In this section, we conduct a series of experiments to analyze the suitability of the proposed system. Note that the goal of the experiments is not to demonstrate that we outperform the rest of the methods in terms of accuracy, as improving the quality of the person detector is not one of our main contributions. As stated in the introduction, the contributions of this work are focused on overcoming the limitations of the existing alternatives regarding the multi-camera scenarios, the monitoring range, and the scalability. Furthermore, we also present an experiment comparing the proposed 3D cylinder estimation procedure with other alternative 3D projection methods.

The server used to carry out the experiments is equipped with an NVIDIA Tesla V100 GPU and an Intel Xeon Gold 6230 CPU. Regarding the programming language, the whole system is implemented using C++.

### System performance

The accuracy of the tasks mentioned in Section 3.8 is related to the correct detection and tracking of all the individuals in the scene, and their position being accurately estimated. For this reason, to evaluate the performance of the proposed system, we focus on measuring the quality of the tracks and the accuracy of the positioning.

To measure the quality of the generated tracks, we use the metrics described in [15], developed to precisely compare different multi-object tracking methods in crowded scenes:

- Multi-object tracking accuracy (*MOTA*): evaluates the tracker performance combining the information of three sources of errors (false negatives, false positives and ID swaps).
- Mostly tracked (*MT*), partially tracked (*PT*) and mostly lost (*ML*) tracks: A target is mostly tracked if it is successfully tracked for at least 80% of its life span; and it is considered as mostly lost if it has been tracked for less than 20% of its total length.
- Number of fragmentations (*FM*): counts how many times every ground truth trajectory is interrupted (untracked).
- Fragmentation ratio (*FR*): relative number of fragmentations (FM/Recall).

Furthermore, to measure the accuracy of the positioning we adopt the following metrics:

- Precision (*P*): measures the reliability of the detections taking into account the true positives over the total positives.
- Recall (*R*): percentage of detected targets over the total number of targets.
- F1 Score (*F*1): harmonic mean of the precision and recall:
  2 $$ F1 = 2\frac{P\cdot R}{P+R} $$
- Average Positioning Error (*APE*): average difference in meters between the ground truth and the estimated 3D feet positions of the people in the scene.

**Fig. 10:**
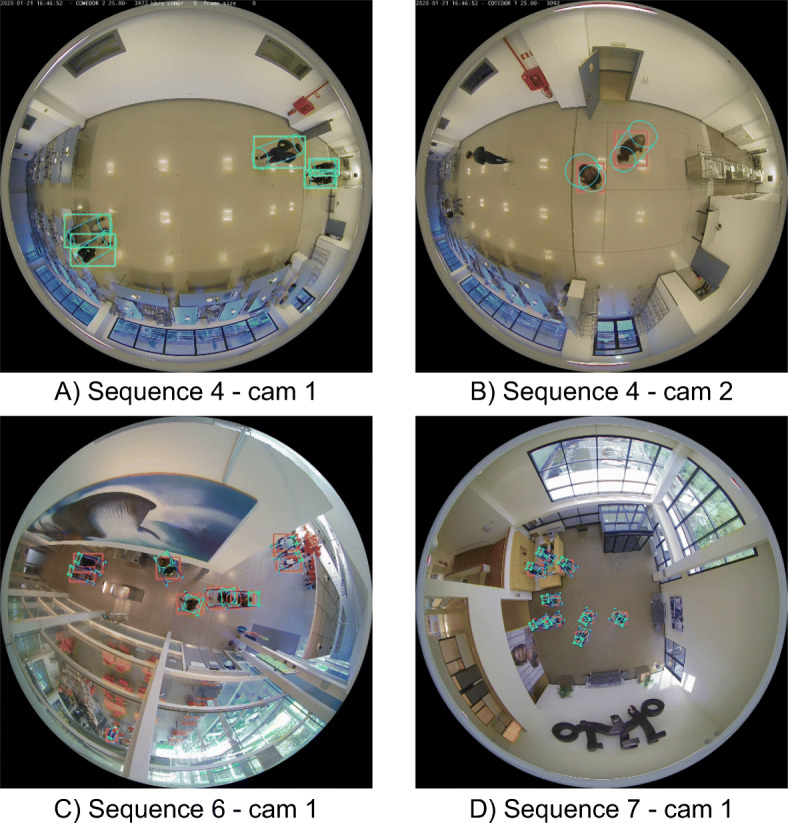
Several examples of the proposed evaluation sequences

To evaluate the system, we consider using different public datasets [18, 32]. Nevertheless, none of them provides the intrinsic and extrinsic parameters of the involved cameras, which are necessary for the 3D projection step. Furthermore, the available datasets only cover single-camera scenarios with very limited monitoring areas. Therefore, we generate 7 sequences with different scenarios, number of cameras, heights, number of identities and levels of occlusion. The details of each sequence are presented in the Table 1. In addition, some examples of the different sequences are shown in Fig. 10.

**Table 1:** Details of the different sequences considered for the system evaluation

Seq	Sc	NC	Heights	Rad	Frame	IDs	Occ
1	1	2	3.3, 3.3	3.5	1610	3	1
2	1	2	3.3, 3.3	3.5	654	5	2
3	1	2	3.3, 3.3	3.5	750	5	3
4	1	2	3.3, 3.3	3.5	1083	4	4
5	1	2	3.3, 3.3	3.5	837	5	5
6	2	1	5.5	8	334	6	2
7	3	1	8.1	10	578	13	4

For each sequence we specify the scenario, number of cameras, camera heights in meters, radius of the monitored area for each camera in meters, number of frames, number of identities, and occlusion level (from 1 to 5)

For each sequence we manually annotate the identity and 3D center point on the ground plane (feet point) of all individuals. With this ground truth we extract the selected metrics. For each sequence, a monitoring region is defined. If a subject abandons this region and enters again it is considered as a new track. Therefore, the number of tracks may be greater than the number of identities. For multi-camera sequences we also evaluate the system for each camera separately to be able to analyze the impact of the multi-camera fusion procedure. The results are shown in Table 2.

**Table 2:** Results of the proposed method in the defined evaluation sequences

Seq	Cam ID	MOTA	MT	PT	ML	FRAG	FRAG ratio	P	R	F1	APE
1	1,2	1.000	8	0	0	0	0.000	1.000	1.000	1.000	0.094
1	1	1.000	7	0	0	0	0.000	1.000	1.000	1.000	0.089
1	2	1.000	8	0	0	0	0.000	1.000	1.000	1.000	0.069
2	1,2	0.994	12	0	0	0	0.000	0.994	1.000	0.997	0.151
2	1	1.000	9	0	0	0	0.000	1.000	1.000	1.000	0.071
2	2	0.993	11	0	0	0	0.000	0.993	1.000	0.997	0.063
3	1,2	0.965	5	0	0	6	0.061	0.998	0.977	0.987	0.136
3	1	0.973	9	0	0	8	0.081	0.999	0.984	0.991	0.084
3	2	0.966	10	1	0	2	0.021	0.999	0.975	0.986	0.077
4	1,2	0.966	5	0	0	6	0.061	0.983	0.989	0.986	0.155
4	1	0.997	25	0	0	1	0.010	1.000	0.999	0.999	0.085
4	2	0.983	22	1	0	6	0.061	1.000	0.986	0.993	0.095
5	1,2	0.975	10	0	1	3	0.031	0.999	0.984	0.991	0.186
5	1	0.952	7	1	0	11	0.115	0.999	0.957	0.978	0.097
5	2	0.991	12	1	0	1	0.010	1.000	0.993	0.996	0.085
6	1	0.982	6	0	0	3	0.031	0.999	0.983	0.991	0.089
7	1	0.935	13	1	0	15	0.157	0.986	0.957	0.971	0.112

The considered metrics, described in Section 4.1, measure the quality of the generated tracks and the accuracy of the positioning. For multi-camera sequences, we also run the system for each camera separately

From Table 2 it can be observed that, even in sequences with a high level of occlusions, the quality of the tracks (measured by the *MOTA* metric) always remains above 90%. In the sequences with more than one camera, the achieved *MOTA* values are very close to those obtained by processing the cameras separately. This highlights the high 3D precision obtained by 3D cylinders estimation and fusion, which allows merging detections from different views using only the lower center point of the cylinder. Furthermore, in some cases (e.g. in sequence 5), the *MOTA* values obtained in the multi-camera sequences outperform the ones from the separate cameras. This is thanks to the multi-camera fusion procedure, where the detection errors of one camera can be corrected with the information of other overlapped cameras.

**Fig. 11:**
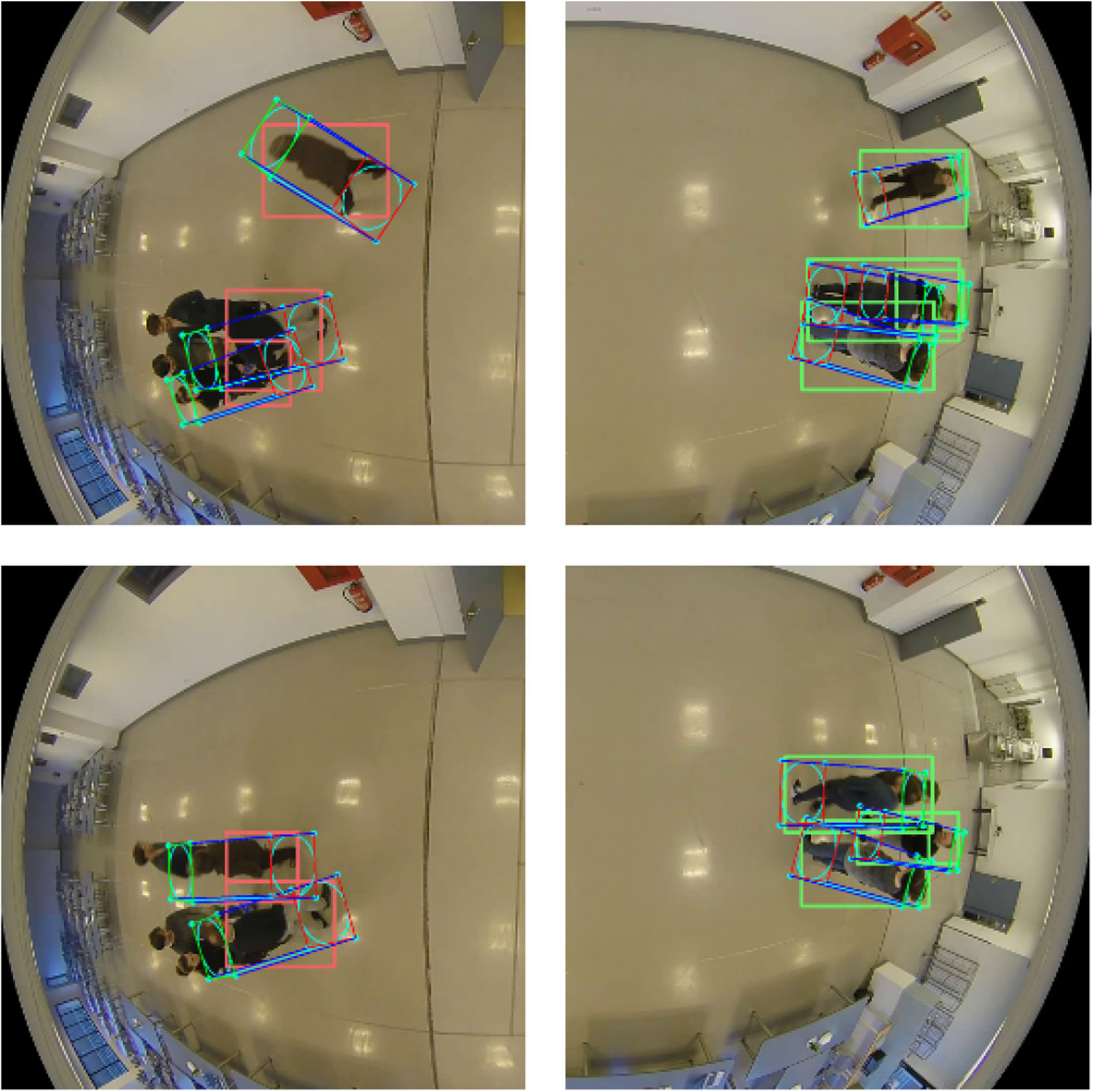
Examples of occlusions in sequence 5. People occluded in the areas furthest from the camera are detected by the complementary camera. Thus, the number of fragmentations is reduced and the functional area of each camera is increased

Apart from the *MOTA* metric, the robustness of the tracks is evident by the reduced number of fragmentations. Even for sequence 5, with more than 800 frames and a high level of occlusions (see Fig. 11), only 3 fragmentations occur when the two cameras are processed together. People occluded in the furthest areas from the camera are detected by the complementary camera and vice versa. Thus, the number of fragmentations is reduced and the functional area of each camera is increased.

On the other hand, attending to the metrics considered to evaluate the precision of the positioning, it can be observed that in all the scenarios an F1 Score higher than 97% is obtained, which means that there are hardly any false positives and negatives even in the sequences with a high level of occlusions. Thus, the system is able to successfully estimate the occupancy of the monitored area. As for the *APE*, for sequences with a single camera it remains below 10 centimeters, while for multi-camera sequences it increases to 15-19 centimeters. This is because the calibration between cameras is not perfect and the positions estimated for the same detection from different cameras do not exactly match.

### 3D projection performance

In this section we present an experiment comparing the performance of the proposed method using three different 3D projection approaches:

- Projecting the center of the detection bounding box. This is the approach followed in the related works mentioned in Section 2.1 [4, 6, 26, 30, 43].
- Projecting the point of the bounding box closest to the center of the image. As mentioned in Section 3.5, for cameras with fish-eye lenses, the furthest point from the camera of a vertical object (i.e. the feet position of a person) corresponds to the object point closest to the center of the generated image (see Fig. 6).
- Estimating the 3D cylinder. This is the proposed approach presented in Section 3.5.

**Fig. 12:**
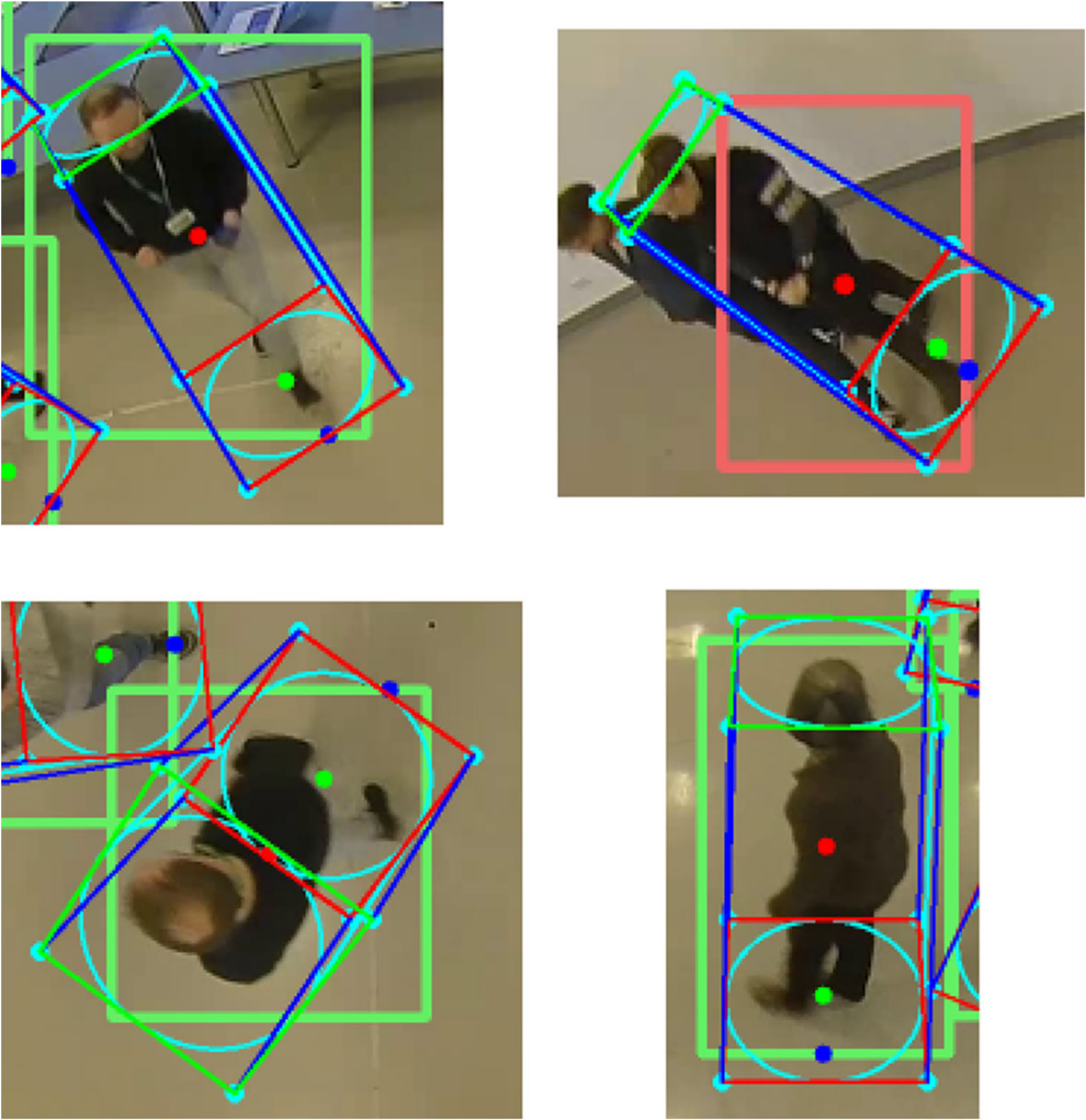
Several examples of the positions estimated by the 3D projection approaches compared in Section 4.2. The red point corresponds to the center of the detection bounding box, the blue point to the point closest to the center of the image, and the green point to the proposed method

We repeat the previous experiments for each projection method. The results of the experiments are presented in Table 3. In this table, we compare the metrics for measuring the positioning accuracy: precision (*P*), recall (*R*), F1 score (*F*1) and average positioning error (*APE*). It can be observed that the central point approach notably achieves the worst results. In addition, compared to the other approaches, with this method the number of false positives and negatives increases, reflected in the decrease in precision and recall. Finally, if we compare the other two methods, the proposed approach achieves the best result by far, reducing the average positioning error by more than 4 times in most cases. It can be observed that, when using the closest point to the image center, the precision for multi-camera sequences worsens. These false positives are caused because the positioning using this method is not accurate enough to fuse the detections of the overlapped cameras. Several examples of the positions estimated by the evaluated approaches are shown in Fig. 12.

**Table 3:** Comparison of the performance of the proposed method using different 3D projection approaches: projecting the center of the bounding box; projecting the point closest to the center of the image; and estimating the 3D cylinder (proposed approach)

S	C	Bounding Box Center				Closest Point to Image Center				3D Cylinder Lowest Center			
		P	R	F1	APE	P	R	F1	APE	P	R	F1	APE
1	1,2	0.801	0.753	**0.776**	**0.600**	0.717	1.000	**0.835**	**0.216**	1.000	1.000	**1.000**	**0.094**
1	1	0.849	0.653	**0.738**	**0.612**	1.000	1.000	**1.000**	**0.215**	1.000	1.000	**1.000**	**0.089**
1	2	0.893	0.710	**0.791**	**0.453**	1.000	1.000	**1.000**	**0.273**	1.000	1.000	**1.000**	**0.069**
2	1,2	0.827	0.732	**0.776**	**0.572**	0.818	0.995	**0.898**	**0.388**	0.994	1.000	**0.997**	**0.151**
2	1	0.687	0.445	**0.540**	**0.618**	1.000	1.000	**1.000**	**0.251**	1.000	1.000	**1.000**	**0.071**
2	2	0.809	0.463	**0.589**	**0.526**	1.000	1.000	**1.000**	**0.355**	0.993	1.000	0.997	**0.063**
3	1,2	0.765	0.705	**0.734**	**0.481**	0.840	0.970	**0.900**	**0.301**	0.998	0.977	**0.987**	**0.136**
3	1	0.880	0.476	**0.617**	**0.524**	0.987	0.982	**0.985**	**0.287**	0.999	0.984	**0.991**	**0.084**
3	2	0.892	0.627	**0.736**	**0.481**	0.998	0.972	**0.985**	**0.326**	0.999	0.975	**0.986**	**0.077**
4	1,2	0.767	0.741	**0.754**	**0.471**	0.798	0.986	**0.882**	**0.297**	0.983	0.989	**0.986**	**0.155**
4	1	0.895	0.622	**0.734**	**0.493**	0.990	0.998	**0.994**	**0.268**	1.000	0.999	**0.999**	**0.085**
4	2	0.763	0.556	**0.643**	**0.455**	0.978	0.985	**0.982**	**0.304**	1.000	0.986	**0.993**	**0.095**
5	1,2	0.873	0.751	**0.807**	**0.504**	0.860	0.970	**0.912**	**0.395**	0.999	0.984	**0.991**	**0.186**
5	1	0.903	0.505	**0.647**	**0.569**	0.997	0.945	**0.971**	**0.258**	0.999	0.957	**0.978**	**0.097**
5	2	0.811	0.484	**0.606**	**0.521**	0.992	0.994	**0.993**	**0.330**	1.000	0.993	**0.996**	**0.085**
6	1	0.907	0.830	**0.867**	**0.331**	0.978	0.974	**0.976**	**0.332**	0.999	0.983	**0.991**	**0.089**
7	1	0.927	0.888	**0.907**	**0.474**	0.982	0.953	**0.967**	**0.272**	0.986	0.957	**0.971**	**0.112**

S stands for the sequence number and C for the camera IDs involved in the test

## Discussion

In this section we want to analyze the results obtained in the experiments presented on the previous section and highlight the strengths and weaknesses of the proposed method, comparing it with other approaches mentioned in the related work. In [6] and [4], the authors declare that they use overhead datasets for training and testing. However, we were unable to find these datasets available online for comparison. The rest of the related works use images with side or frontal perspectives, so it is not possible to make a fair comparison with them. Furthermore, none of the test datasets they use include the necessary calibration parameters to be able to carry out the projection. Finally, none of the mentioned methods is publicly available, so we cannot make a comparison using our test sequences either. For this reason, we focus on comparing the proposed projection method with the projection method used in all other related works. All the mentioned methods that perform projection [4, 6, 26, 30, 43] use the central point of the bounding box to estimate the position of the subject. As commented in the previous section, from the results of Table 3, it can be observed that our method outperforms the the method based on central point projection, decreasing the average positioning error by more than 4 times in most of the cases.

Main drawback is that estimating the 3D cylinder requires additional computing time. More specifically, for the hardware used in the evaluation (Intel Xeon Gold 6230), the estimation time for each cylinder is 0.6 ms. It is a despicable amount of time but it grows in proportion to the number of detections. Nevertheless, this procedure would be easily parallelizable using multi-threading on CPU or GPU, since the estimation of each cylinder is independent of the rest.

Regarding the size of the monitored area, in [6] and [4] the authors do not give any details about this topic. Analyzing the data and images provided in the articles, we estimate that they cover an area with an approximate radius of 5 meters for a fisheye camera placed 6 meters high. If we compare this setup with the one of our evaluation sequence 6 (see Table 1), it can be observed that, even though the camera is positioned at a lower height, the radius of the area covered (8 meters) by our method is much higher.

Another strong point of the proposed method is its ease of adaptation to new environments. As we discussed in previous sections, the system is capable of working with cameras positioned at different heights, thanks to the fact that the fusion of multi-camera detections and tracking is carried out taking into account the position in real coordinates and not the 2D bounding box of the detection. This makes the method adaptable to any indoor environment with the only requirements that there is a ceiling where to place the cameras, that the cameras are placed perpendicular to the ground plane and that there is a small overlap region between two consecutive cameras.

Finally, the proposed system is also easily scalable, allowing new cameras to be added at any time. The newly added cameras only need to be calibrated to obtain the intrinsic and extrinsic parameters referenced to the coordinate origin of the global system.

## Conclusions

The aim of this work was to create a system capable of monitoring people in large infrastructures, especially to guarantee compliance with the health measures imposed by COVID-19. In order to algorithmically assess compliance with some of these measures, such as maintaining social distance, a precise position estimation of the subjects is necessary and complete occlusions must be avoided. Therefore, we decided to tackle the problem using people detection from an overhead perspective. This is yet an unexplored topic and the few solutions proposed only work in single camera scenarios and cover a very limited area.

To overcome these limitations, we present a multi-camera BEV people flow monitoring system, capable of extracting reliable real-time performance indicators in extremely large infrastructures, such as airports or shopping centers. The proposed system breaks with the traditional pipeline, applying the projection step just after the detection stage. This modification allows tracking the subjects uninterruptedly all over the monitored area using just a single tracker instance and using multiple cameras installed at different heights. Furthermore, we present a novel 3D projection and multi-camera fusion procedure. It estimates the best fitting 3D cylinder for each detected bounding box and fuses the cylinders of the overlapping regions of the camera views that belong to the same person. This corrects possible occlusion problems and allows us to expand the useful range of the cameras.

Conducted experiments, presented in Section 4, demonstrate that the proposed system is suitable for real-time sanitary-measures-control applications, such as guaranteeing a safe interpersonal distance, respecting the indoor capacity limitations, identifying the most crowded time intervals or tracking subjects who violate the established measures. Furthermore, the proposed projection approach achieves an average positioning error below 0.2 meters, with an improvement of more than 4 times compared to other methods.

Future work will focus on extending the application of the system to other tasks such as subject re-identification. For this task, the system should be able to fuse the information of overhead and frontal cameras in order to identify the subject using facial recognition and track it throughout infrastructure. The strengths of the proposed method lie in its ability to monitor large areas in an efficient and scalable manner. It could be used in other applications that require this capacity, such as for monitoring traffic in cities or dangerous vehicles that have committed an infraction. We also think that it suitable for monitoring certain sports in which the playing field is very wide, such as football or rugby, both for tracking the players and the ball. Furthermore, we will also work on improving the accuracy of the detection network to reduce the number of false positives and negatives and improve the performance of the entire system. Finally, we will also focus on improving the data analytics logic to incorporate also temporal information for the distance monitoring, using the computed subject trajectories.
